# Combined Sacroplasty and Iliosacral Fixation Using Triangular Titanium Implants for the Treatment of Sacral Insufficiency Fractures with Concomitant Sacral Instability

**DOI:** 10.7759/cureus.1351

**Published:** 2017-06-14

**Authors:** Ryan Johnson, Jason Seibly

**Affiliations:** 1 Graduate Medical Education, Advocate Bromenn Medical Center; 2 Neurosurgery, Central Illinois

**Keywords:** sacrum, insufficiency fracture, sacroplasty, iliosacral fixation

## Abstract

Sacral insufficiency fractures (SIFs) are a common, under-recognized cause of debilitating low back pain, particularly in elderly patients with osteoporotic bone disease or risk factors for bone demineralization. Conservative therapy has been the mainstay of treatment for these types of fractures. This places patients at increased risk for the development of secondary illnesses associated with prolonged immobility, which might prevent a full return to the preinjury level of function. Surgical intervention for SIFs has increased over the past two decades in an attempt to overcome these complications and improve patient functionality. Sacroplasty and iliosacral screw stabilization are two specific procedures performed to treat SIFs. The purpose of this case report is to further document the existence of this condition, as well as to detail a novel approach for surgically treating this condition with a combination of the above procedures.

## Introduction

Sacral insufficiency fractures (SIFs) are a common, under-recognized cause of debilitating low back pain [1-2], particularly in elderly patients with osteoporotic bone disease or risk factors for bone demineralization. Until recently, the treatment for SIFs was mainly conservative, including bed rest, oral analgesic medication, and physical therapy and rehabilitation. It is well-known that prolonged bed rest increases the risk of deep venous thrombosis, pulmonary embolism, pneumonia, cardiac dysfunction, and muscle atrophy [1,3]. Surgical techniques to treat SIFs do exist. Sacroplasty, similar to vertebroplasty, has emerged as a technique that can relieve the pain caused by SIFs and that allows the patient to return to a higher quality of function. Iliosacral screw stabilization has also been described in the literature to treat refractory pain in patients with SIFs [4]. We present the case of an elderly female with a documented SIF, whose lower back pain and mobility improved significantly after a novel approach combining these two surgical principles. This resulted in an immediate reduction in her pain and allowed her to return more quickly to ambulatory status and eventually regain her preinjury level of function.

## Case presentation

**Figure 1:**
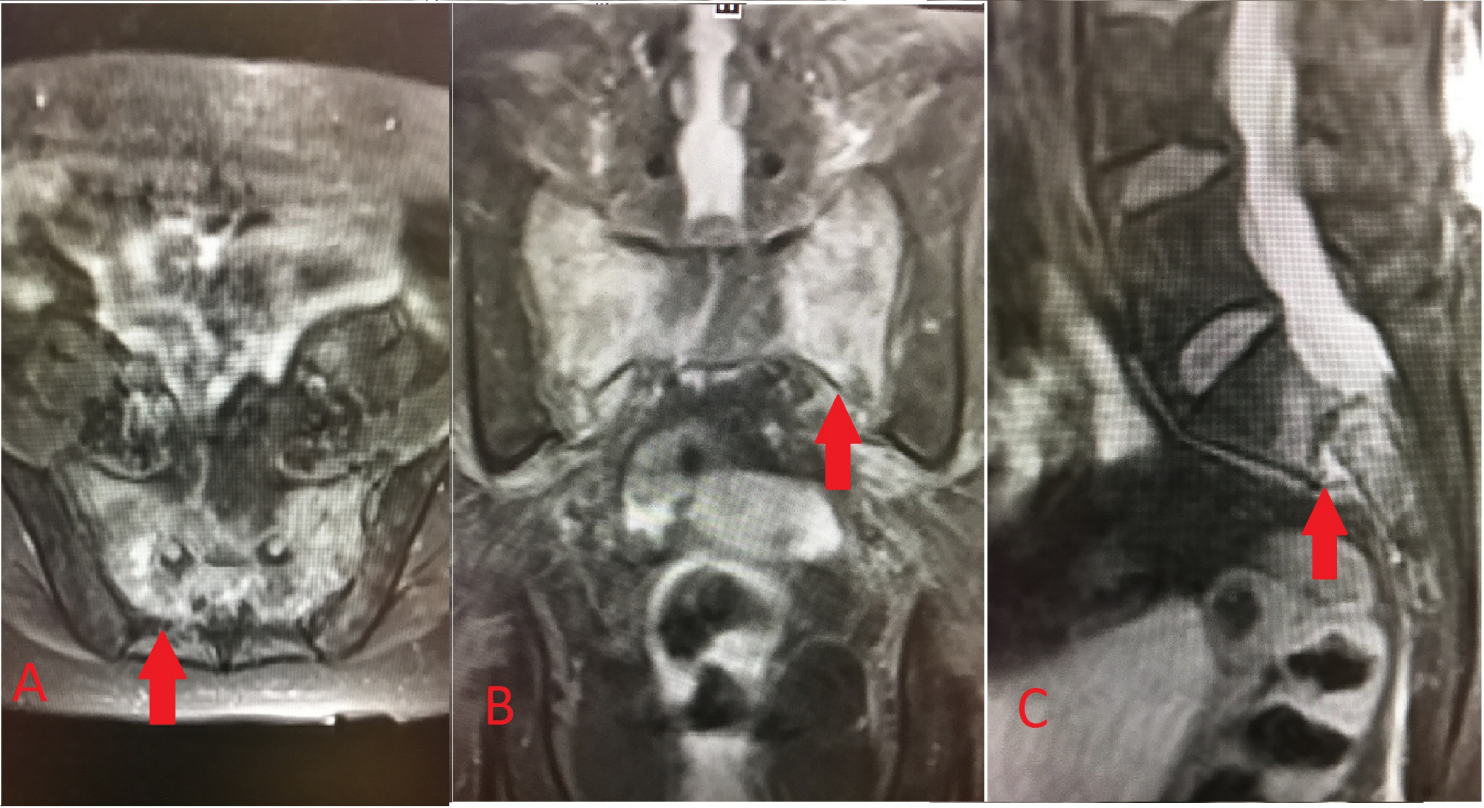
MRI of the sacrum and pelvis A. Axial T2-weighted (T2W) short tau inversion recovery (STIR) sequence demonstrating hyperintensity (arrow) located within the bilateral sacral ala and the body of the sacrum; B. Coronal T2W STIR; C. Sagittal T2W STIR again demonstrating hyperintensity but also showing  S1-S2 anterolisthesis (arrow)

**Figure 2:**
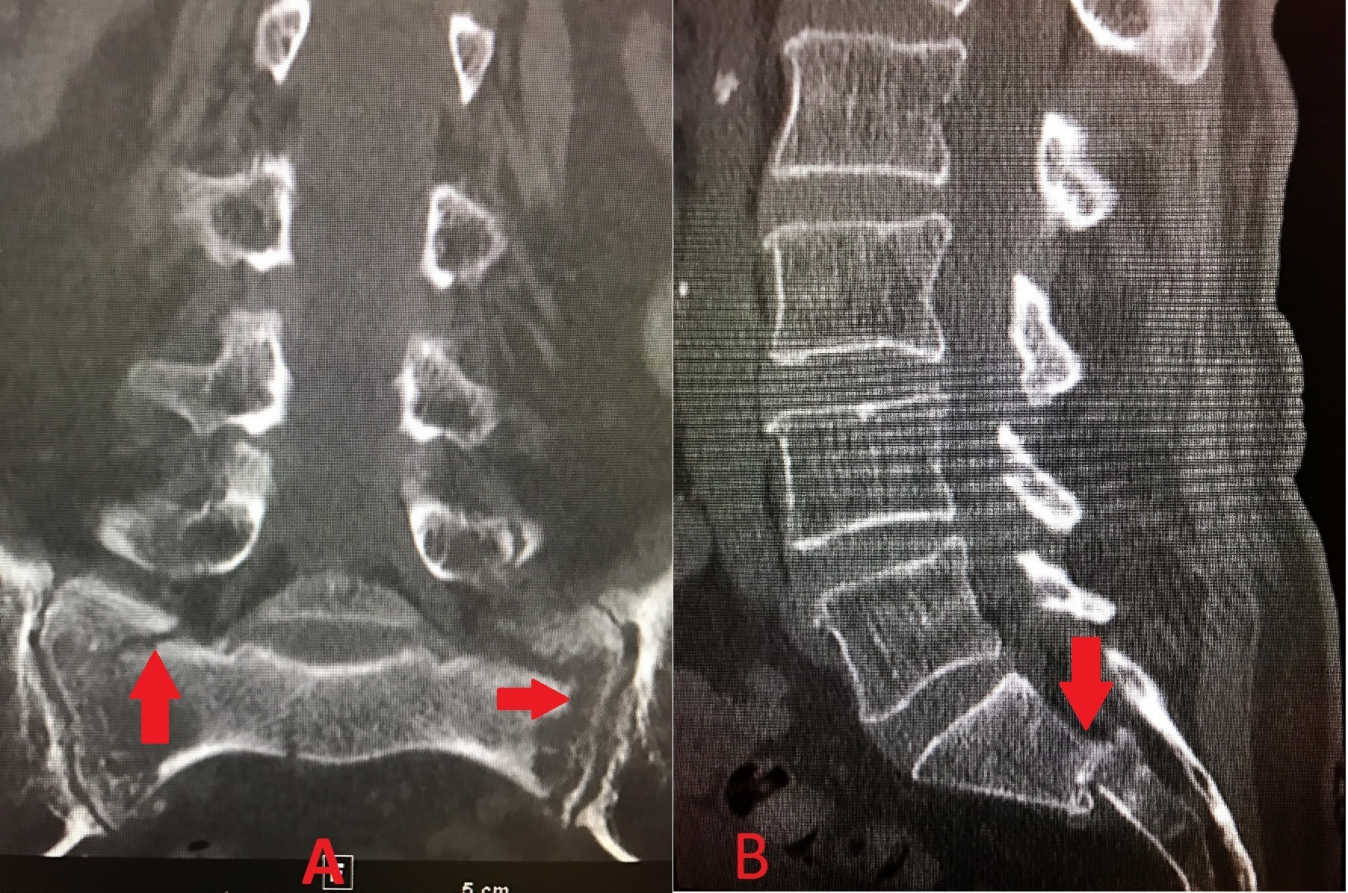
CT of the lumbar spine, sacrum, and pelvis A. Coronal view showing comminuted SIFs (arrows) through the bilateral sacral ala; B. Sagittal view showing S1-S2 anterolisthesis (arrow)

**Figure 3:**
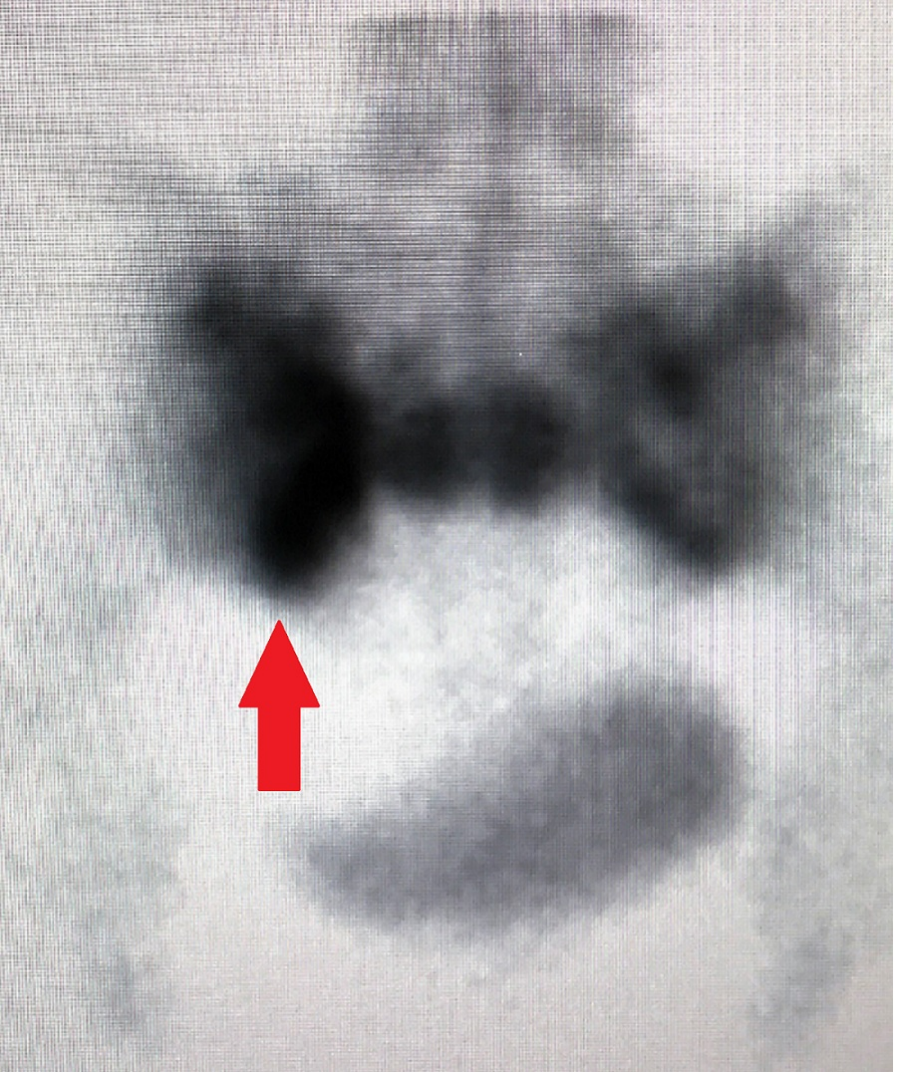
Technitium-99m labeled bone scintigraphy Increased uptake of technitium-99m, demonstrated by the arrow, in the bilateral sacral ala and across the body of the sacrum in the characteristic "Honda logo" shape

An 84-year-old Caucasian female with a past medical history of hypertension, hypothyroidism, and chronic low back pain presented for an exacerbation of her low back pain. Before her hospitalization, she had been managing her low back pain with opioid analgesics and physical therapy at a skilled nursing facility for the prior four weeks. She was scheduled to see Neurosurgery in the outpatient setting, but her back pain became more severe. It progressed to the point that she could no longer sit nor ambulate without being in severe pain. Her back pain worsened with any movement, particularly while flexing forward, and radiated down the posterior aspect of the right leg in the S1 dermatome. She denied any numbness or tingling, and no saddle anesthesia or any acute bowel or bladder changes were noted. The extent of pain was truly debilitating, requiring hospitalization. She underwent a workup that included a magnetic resonance imaging (MRI) scan of the lumbar spine, which did not reveal any significant finding, and an MRI of the abdomen and pelvis, which revealed a severe bilateral SIF with grade 2 anterolisthesis of S1-S2 (Figure 1A-1C). A subsequent computed tomography (CT) scan revealed bilateral L5 transverse process fractures, diffuse bone demineralization, and comminuted SIFs through the bilateral sacral ala with malalignment at the S1-S2 level (Figure 2A-2B). Bone scintigraphy (Figure 3) was obtained of the bony pelvis, which demonstrated an H-shaped increased uptake in the bilateral sacral ala and the anterior aspects of the sacrum. This situation would typically disable a patient and, in this age group, likely contribute to mortality. Surgical intervention was recommended to prevent further debilitation and aid in a return to former ambulatory status. Following the surgical intervention, she could ambulate on postoperative Day 1, with only mild discomfort. She returned to the skilled nursing facility to finish her rehabilitation and regained most of her preinjury level of function. She was ambulating independently and with no pain at her six-week postoperative follow-up. She experienced no complications from the surgery, nor did she experience the common complications of prolonged immobility associated with SIFs.

 

### Surgical technique

The surgical decision-making was complex, and several factors had to be considered. First, the author did not feel a sacroplasty was sufficient to fixate the sacral alar fractures and address the S1-S2 unstable anterolisthesis. Additionally, although commonly performed, sacroplasties are not FDA-approved and are considered “investigational.” Iliosacral screw fixation is another surgical technique to treat sacral fractures. The author was concerned about the patient’s bone quality and about the fact that a simple screw fixation would not be strong enough to stabilize the fractures adequately. With bilateral alar fractures in the sagittal plane and a dislocation of S1-S2 in the horizontal plane, the surgeon was looking for the strongest construct possible; yet, he wanted a less-invasive option to minimize surgical time and morbidity. 

The lead author decided to perform a bilateral iliosacral fixation using the triangular titanium implants (iFuse implants from Si-BONE, San Jose, CA) commonly used for minimally invasive sacroiliac (SI) joint fusions. The shape of these implants is felt to be superior to standard threaded screws. The design of these implants also allows for the passage of a kyphoplasty needle (Kyphon Express from Medtronic, Dublin, Ireland) through the central cannulation. This permits the direct injection of polymethylmethacrylate (PMMA) through the iFuse implant into the sacral ala, providing additional fracture augmentation and implant strength.

**Figure 4:**
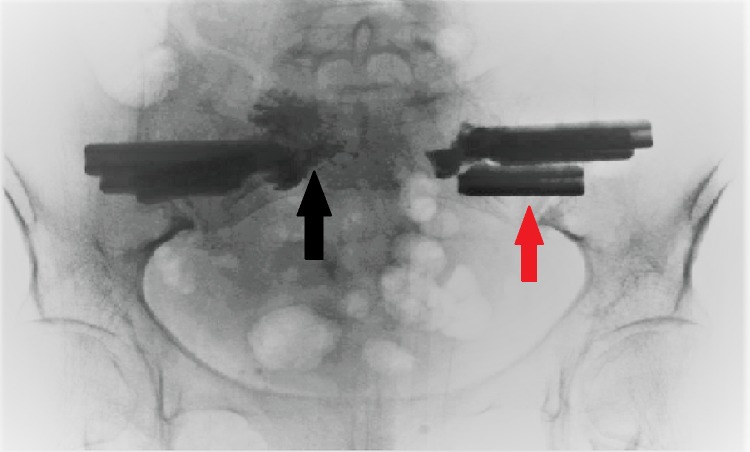
Intraoperative C-arm fluoroscopic radiograph of the pelvis and sacrum The figure shows three triangular implants (red arrow) placed bilaterally through the ilium and sacral ala across the SI joint. Also seen is the polymethylmethacrylate (PMMA) cement (black arrow) inserted into the SI joint bilaterally.

The patient was positioned in the standard prone position on a Jackson operating table. She was prepped from hip to hip. A C-arm was used to mark the one-inch skin incision bilaterally, following the posterior sacral line on lateral fluoroscopy. After the skin had been incised, Steinmann pins were placed through the lateral ilium across the SI joint and into the sacral ala. After each pin had been placed, a three-dimensional image was obtained using the O-arm (Medtronic, Dublin, Ireland) to assess the pin position and make sure it crossed the alar fracture line. Next, the drill-guide was placed over the pin, resting on the lateral ilium. The implant length was then measured, and the implant trajectory was drilled over the pin. After the drilling, a triangular channel was created using the implant broach. The iFuse implant (Si-BONE, San Jose, CA) was then inserted and tapped into position. Three implants were placed on each side. Two implants were intentionally placed above the S1-S2 interspace, and the third inferior implant was placed below the transverse fracture line. After the implants were placed, a Kyphon Express needle (Medtronic, Dublin, Ireland) was placed through the central cannulation of each implant. Approximately 2 ml of PMMA was injected into the medial ala through each implant under live fluoroscopy. Several times during the cement delivery, a C-arm radiograph was obtained to make sure the cement did not undergo extravasation nor violate the S1 neuroforamen. This created a solid construct, fixating the sacrum securely to the ilium. The intraoperative C-arm fluoroscopic radiograph is shown in Figure 4.

## Discussion

SIFs are a common, yet under-recognized, cause of debilitating low back pain in elderly patients [1-2]. First described in 1982 by Lourie [5], SIFs are increasingly diagnosed, but often after a delay because of the relatively nonspecific nature of the clinical symptoms or the potential overlap with a lower lumbar spine pathology. Patients commonly present with vague lower back pain that may radiate into the buttock, hip, groin, or lower extremities because of a lower lumbar or sacral radiculopathy [6]. It is estimated that neurologic symptoms occur in 5%-6% of patients with SIFs; the probability of neurologic injury is associated with the different anatomic zones that can feature SIFs, as classified according to the Denis Classification, with Zone III having the highest probability of neurologic injury [6-7]. The cauda equina syndrome secondary to a SIF has also been described in the literature [8].

Osteoporosis is a well-known risk factor for vertebral body and long bone fractures in the elderly population, both men and women. As such, SIFs are more common in the elderly suffering from osteoporosis. Conditions that result in bone demineralization and the subsequent formation of secondary osteoporosis would also increase the chance of developing SIFs. These conditions include Cushing’s syndrome/disease, hyperparathyroidism, renal osteodystrophy, Paget’s disease, prolonged immobilization, and a history of malignancy with prior pelvic radiation [6]. Stress fractures are classified into two categories, fatigue and insufficiency fractures. Fatigue fractures occur when abnormal stress is applied to a bone with normal elastic resistance, while insufficiency fractures occur when physiological stress (such as normal ambulation) is applied to a bone with deficient elastic resistance [6,9]. Wolff’s Law states that the compressive strength of the trabecular bone is proportional to the square of its density [9]. Osteoporotic bones result in a decrease in density, consequently decreasing their compressive strength exponentially and leading to an increased risk of fracture with physiologic loads [9].

The in-depth imaging characteristics of SIFs are beyond the scope of this case report. The author would like to refer readers to a review article published in the *American Journal of Neuroradiology* in 2010 by Lyders and colleagues [6]. The review article indicates the high frequency of associated fractures in patients with SIFs, specifically pelvic insufficiency fractures of the pubic rami and parasymphyseal region (88%) [6]. MRI and bone scintigraphy are the two most-sensitive imaging modalities for this condition, with sensitivities approaching 100% and 96%, respectively [6]. Each imaging modality has its limitations, which could result in a misdiagnosis. In patients with a history of cancer, the concern for metastatic disease results in biopsies to the region of increased uptake on bone scintigraphy. The classic appearance on bone scintigraphy is the uptake of the technetium in an “H” pattern, indicating bilateral vertical fractures and a transverse fracture. However, this “Honda sign” is only seen in 20%-40% of patients with SIFs [6].

In our patient, hyperintensities were noted diffusely in the bilateral sacral ala and the S1 and S2 regions on the MRI T2W STIR sequence, consistent with acute fracture and inflammation in the affected areas (Figure 1A-1C). Additionally, a CT scan of the pelvis demonstrated bilateral comminuted SIFs with S1-S2 anterolisthesis and bilateral L5 transverse process fractures (Figure 2A-2B). An MRI is not the most accurate test for bone fractures, which is where a CT scan plays a role. Our patient additionally underwent bone scintigraphy, which demonstrated increased uptake in the characteristic “Honda logo” shape (Figure 3). The authors felt that the patient’s clinical evaluation, in conjunction with MRI/CT/bone scintigraphy imaging of the pelvis and sacrum, were satisfactory for the diagnosis of SIF and subsequent surgical planning.  

Until recently, the treatment for SIFs was conservative, including prolonged bed rest, rehabilitation, and analgesic medications. The problems that arise secondary to conservative management include an increased risk of deep venous thrombosis, pulmonary embolism, cardiovascular dysfunction, muscle atrophy, decubitus ulceration, and pneumonia [1,3]. Newer modalities in treatment, including sacroplasty and iliosacral screw fixation, have emerged to diminish the patient’s pain as well as to allow them to mobilize sooner to prevent the complications listed above. One case series of 24 patients demonstrated an improvement in pain control and mobility after a combination of sacroplasty and a single percutaneous iliosacral screw traversing the bilateral ilia [4]. Multiple case reports demonstrate a significant reduction in the Visual Analog Scale (VAS) for pain after sacroplasty and an earlier return to preinjury functionality. The VAS pain scale before surgery and after surgery was not objectively quantified in our case report. The patient’s severe low back pain was the reason for her debility, with the resultant nonambulatory status. Our unique approach accounts not only for the instability of the S1-S2 anterolisthesis by applying multiple fixation points for increased stability, but also allows for the direct management, via sacroplasty, of the micro-motion thought to be causing the pain associated with the SIFs. The combination of the two resulted in the patient regaining her former independent ambulation status and preinjury function, as well as benefitting from a reduction in her pain.

## Conclusions

SIFs are a common debilitating cause of lower back and sacral pain in elderly patients. Diagnosis and treatment are usually delayed secondary to nonspecific signs and symptoms. MRIs and bone scintigraphy are the most-sensitive imaging modalities, but the utilization of CT as an adjunct imaging study can enhance the clinical picture and help confirm the diagnosis. Treatment is usually conservative at the beginning, and includes prolonged periods of bed rest that could put patients at risk for developing secondary illnesses. Our case report shows the benefit of a combined iliosacral fixation with three titanium implants and sacroplasty. This provided multiple fixation points that increased the stability of the patient’s S1-S2 anterolisthesis. Initially, our patient was nonambulatory. Following surgical intervention, she could ambulate on postoperative Day 1 with only mild discomfort. She returned to the skilled nursing facility to finish her rehabilitation and regained most of her preinjury level of function. She was ambulating independently and with no pain at her six-week postoperative follow-up. Our case report advocates for the utilization of early sacroplasty, iliosacral fixation, or both, with appropriate patient selection, in those patients that are at higher-than-acceptable risk for the complications of conservative management.
